# Applications of Metal Oxide Charge Transport Layers in Perovskite Solar Cells

**DOI:** 10.1002/smsc.202300020

**Published:** 2023-07-27

**Authors:** Jiale Liu, Sheng Li, Anyi Mei, Hongwei Han

**Affiliations:** ^1^ Michael Grätzel Center for Mesoscopic Solar Cells Wuhan National Laboratory for Optoelectronics Key Laboratory of Materials Chemistry for Energy Conversion and Storage of Ministry of Education Huazhong University of Science and Technology Wuhan 430074 Hubei P. R. China

**Keywords:** charge transport layers, metal oxide, perovskite solar cells, SnO_2_, TiO_2_

## Abstract

Metal oxide (MO) charge transport layers (CTLs) are widely used for fabricating highly efficient and stable perovskite solar cells (PSCs) due to their superior stability, material and preparation cost, light transmission, and charge selection. However, the complex surface states, unbalanced carrier mobility, and variable energy band structure determined by MOs can lead to additional interfacial charge recombination and transport losses within the device, which limit further improvements in device performance. Extensive research has been conducted to address these challenges. In this review, an overview of current popular MO‐CTLs and their preparation methods for PSCs are provided. Interface regulation strategies, such as passivating interface defects, modulating interface energy level alignment, and improving interface contact are also discussed, which can enhance the performance of PSCs. Meanwhile, the commonly used dopants and doping strategies for optimizing the charge transport properties of CTLs are also discussed.

## Graphical abstract

Highly efficient and stable perovskite solar cells (PSCs) widely apply metal oxide (MO) charge transport layers (CTLs) due to their superiorities in stability, material and preparation cost, light transmission, and charge selection. Through interface modulation, elemental doping, and morphology control, further breakthroughs in MO‐CTLs‐based device performance can be achieved.

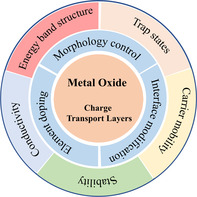

## 1 Introduction

Perovskite solar cells (PSCs) have experienced rapid development in recent years with a significant improvement in power conversion efficiency (PCE) from 3.8% to a certified 25.8% and greatly improved stability.^[^ ^1^ ^]^ The charge transport layers (CTLs), including the electron transport layer (ETL) and the hole transport layer (HTL), are crucial in enhancing performance by constructing selective contacts for charge separation.^[^ ^2^ ^]^ The ETL and HTL materials used in PSCs are expected to possess certain properties, such as appropriate energy level alignment with adjacent layers, high electron/hole mobility and optimal electron/hole concentration, relative transparency, excellent stability, and no reaction with adjacent layers. These properties ensure the rapid charge extraction, reduce nonradiative recombination, block opposite carriers at the interface, and allow light to transport into the absorber layer to be converted, while the material and the interface stability are the cornerstone of long‐lived PSCs. Metal oxide (MO) CTLs are promising candidates for satisfying the above requirements. MO‐CTLs have been developed for decades, and sufficient experience and data have been accumulated. In fact, the rapid development of the performance of PSCs is indispensable to the application of MOs. The most efficient PSCs are based on MO electron transport materials (ETMs) such as TiO_2_ and SnO_2_.^[^ ^3^, ^4^ ^]^ While MO hole transport materials (HTMs) have not achieved the record‐breaking PCEs of organic HTMs, they have shown promising improvements in device stability and reduced material costs.^[^ ^5^, ^6^, ^7^ ^]^

Although MO CTLs have a wide range of applicable conditions including simple preparation process, low cost, good chemical stability, and excellent electrical properties, some issues are still remained to be addressed. On the one hand, the complex surface states of MOs, such as oxygen vacancies, ionic residues, suspended bonds, etc. of MOs can introduce additional nonradiative recombination centers and affect the crystallization and contact of the perovskite layer, which brings negative impact on the device performance and stability.^[^ ^8^, ^9^ ^]^ On the other hand, the unbalanced carrier mobility and low conductivity of the ETLs and HTLs result in interfacial carrier accumulation and resistance loss, leading to severe hysteresis effects, aggravated charge recombination, and transport losses in the device. In addition, the energy band structure of CTLs can be significantly affected by their preparation process and conditions, which can in turn impact their energy band alignment with the perovskite layer, thus limiting further improvements in device performance.

To address these challenges, various strategies have been developed and employed, including morphology control, interface modification, and doping, to achieve better contact and energy level alignment, more stable interfaces, as well as higher carrier concentration and mobility. In this review, we first summarize currently developed MO‐CTLs and their preparation methods. Next, we focus on the PSCs efficiency improvement strategies related to the MO‐CTLs interface. Finally, we summarize doping strategies used to enhance the charge transport capabilities of MO‐CTLs.

## 2 Materials and Deposition Methods for MO‐CTLs

**Figure 1:**
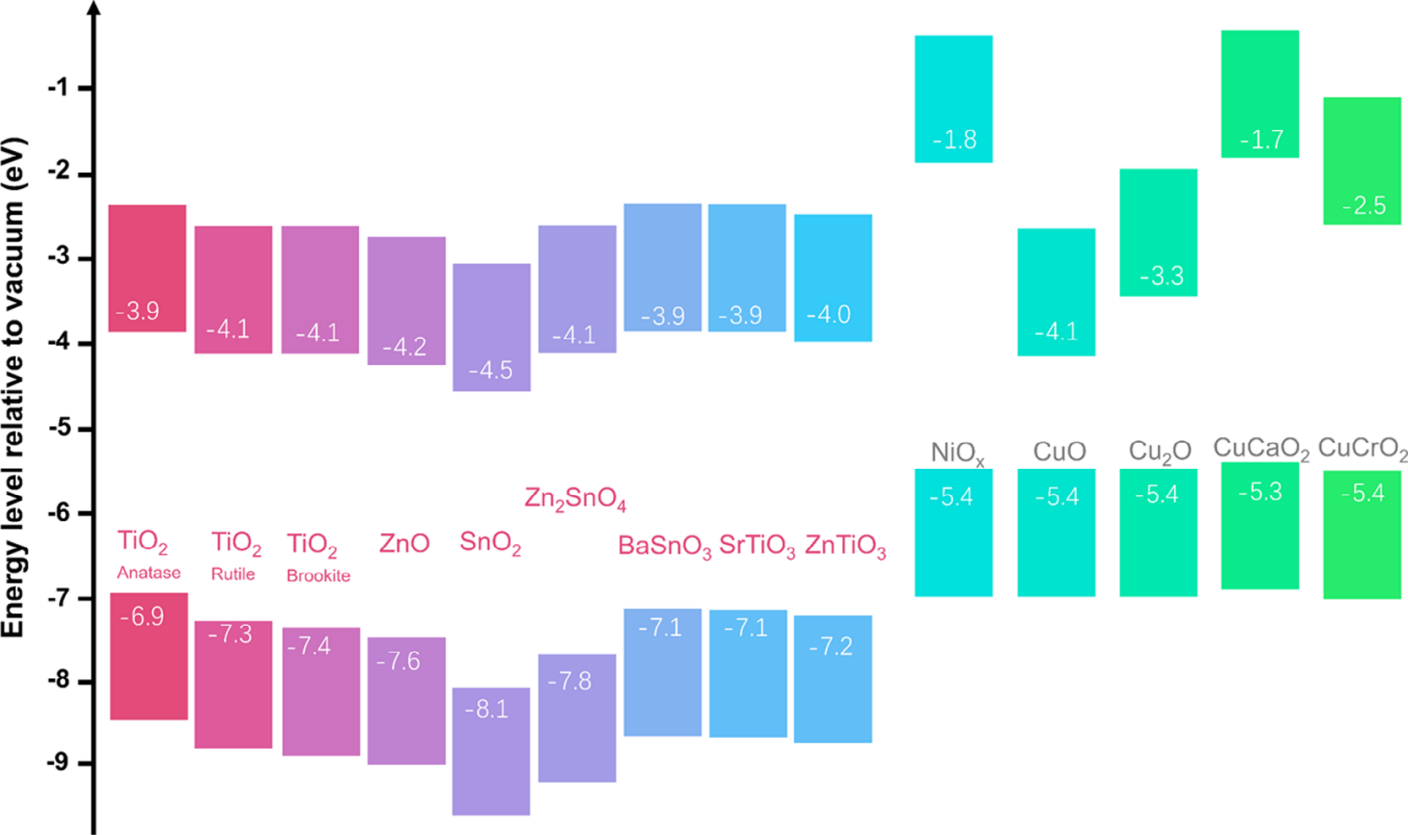
Energy levels of various inorganic ETLs and HTLs.

Various MO‐CTL materials have been developed for PSCs. These materials have varied electric properties and surface properties to meet the requirements of PSCs. The commonly used MO‐CTL materials and their energy level alignments are summarized in **Figure** **1**. The reported ELTs include TiO_2_, SnO_2_, ZnO, BaSnO_3_, ZnSnO_4_, SrTiO_3_, SrSnO_3_, ZnTiO_3_, Nb_2_O_5_, etc., while TiO_2_ and SnO_2_ are the most widely adopted. MO‐ETLs generally have a conduction band minimum level (CBM) that is slightly deeper than that of the perovskite light absorber for extracting photogenerated electrons. At the same time, they have a much deeper valence band maximum level (VBM) than that of the perovskite light absorber for blocking photogenerated holes from perovskite to the negative electrode. Among the various reported MO‐HTL materials that include NiO_*x*_, CuO_*x*_, CuGaO_2_, CuAlO_2_, CuCoO_2_, CuCrO_2_, NiCo_2_O_4_, MoO_*x*_, VO_*x*_, WO_*x*_, CoO_*x*_, etc., NiO_*x*_ and MoO_*x*_ are the most widely used. In contrary to ETLs, HTLs have a slightly higher VBM and a much higher CBM than that of perovskite. These discrepancies allow HTL to extract holes and block photogenerated electrons to the positive electrode. The combination of HTLs and ETLs realizes selective separation of photogenerated charges by extracting and blocking carriers specifically.

CTLs generally possess the planar structure and mesoporous structure, and the preparation methods of MO‐CTLs are diverse. CTLs can be deposited directly from source materials such as via vapor deposition techniques, including atomic layer deposition (ALD) and vapor deposition, and wet processing such as chemical bath deposition (CBD) and spray pyrolysis. These methods are suitable for preparing planar CTLs. In contrast, CTLs can be also deposited indirectly by first preparing CTL nanoparticles through methods such as hydrothermal, sol–gel, and coprecipitation methods. The prepared particles are then dispersed in solvents to form colloids or mixed with organic binders and solvents to form slurries. Generally, the prepared colloids are then adopted for preparing planar CTLs while slurries are generally applied to prepare mesoporous CTLs via spin coating, spray coating, blade coating, or other wet coating methods. The planar CTLs can be prepared at low temperature and adopted for flexible devices, while the mesoporous structure favors charge extraction due to the large contact area. Moreover, the mesoporous CTL has better interfacial stability than planar structures due to the coefficient difference of thermal expansion. From an industrial point of view, the main challenge for planar structure is how to achieve film compactness on large‐area substrates, given that planar CTL is generally only tens of nanometers thick. The mesoporous CTL can be as thick as hundreds of nanometers and is suitable for large‐area film deposition via facile low‐cost technologies such as printing. However, a major obstacle needed to be addressed is reducing energy consumption during the production process, which involves removing organic binders at 400–500 °C to create the pores. Representative research works on preparing CTLs are listed in **Table** **1**.^[^ ^10^, ^11^, ^12^ ^]^

**Table 1:** Summary of devices based on different MO‐CTLs preparation methods

Metal oxide	Formation process and film fabrication	Temp.	Device structure	PCE [%]	Ref
NiOx	Hydrothermal and spin coating	No heating	ITO/NiO_*x*_/Cs_0.05_MA_0.1_FA_0.85_PbI_3_/PCBM/BCP/Ag	23.91	[132]
NiOx	Combustion and spin coating	250	TCO/NiO_*x*_/MA_1−*y*_FAyPbI_3−*x*_Cl_*x*_/PCBM/BCP/Ag	20.2	[133]
NiOx	Chemical bath deposition	500	FTO/mp‐NiO_*x*_/perovskite/PCBM/BCP/Ag	16.7	[134]
NiOx	Electrolysis and electrochemical deposition	300	ITO/NiO_*x*_/MAPbI_3_/PCBM/Ag	19.2	[135]
NiOx	Thermal decomposition and spray pyrolysis	500	FTO/NiO_*x*_/FAPbI_3_/PCBM/TiO_*x*_/Ag	20.65	[136]
NiOx	Screen printing	500	FTO/mp‐TiO_2_/mp‐Al_2_O_3_/mp‐NiO_*x*_/carbon	15.03	[137]
NiOx	E‐beam‐evaporation	RT.	ITO/NiO_*x*_/FAPbI_3_/PCBM/BCP/Ag	23.4	[131]
NiOx	Pulsed laser deposition	200	ITO/PLD‐NiO_*x*_/MAPbI_3_/PCBM/LiF/Al	17.3	[138]
NiOx	Vacuum thermal evaporation	RT.	ITO/NiO_*x*_/MAPbI_3_/C_60_/SnO_2_/Ag	16.64	[139]
CuOx	Oxidation treatment and spin coating	100	ITO/Cu_2_O/MAPbI_3_/PCBM/Ca/Al	13.35	[140]
CuOx	Chemical bath and chemical bath deposition	170	ITO/Cu_2_O/MAPbI_3_/PCBM/Al	8.23	[141]
CuOx	Thermal decomposition and electrospray	500	ITO/CuO_*x*_/MAPbIxCl_3−*x*_/C_60_/BCP/Ag	5.83	[142]
CuCo_2_O_4_	Oxidation treatment and spin coating	350	Ag/C_60_/PCBM/MAPbI_3_/CuCo_2_O_4_/ITO	14.12	[143]
CuAlO_2_	Magnetron sputtering	RT	ITO/a:CuAlO_2_/PEDOT:PSS/MAPbI_3−*x*_Cl_*x*_/PCBM/Ag	14.52	[144]
CuCrO_2_	Hydrothermal and spin coating	150	ITO/c‐CuCrO_2_/MAPbI_3_/PCBM/BCP/Ag	19.0	[145]
Cu_*y*_Cr_z_O_2_	Thermal decomposition and spin coating	200	FTO/CuCrO_2_/MAPbI_3_/PCBM/Ag	17.19	[146]
CuGaO_2_	Hydrothermal and spin coating	100	FTO/c‐TiO_2_/perovskite/CuGaO_2_/Au	18.51	[147]
NiCo_2_O_4_	Co‐precipitation and spin coating	200	ITO/NiCo_2_O_4_/MAPbI_3−*x*_Cl_*x*_/PCBM:C_60_/ZrAcac/Ag	18.23	[148]
NiCo_2_O_4_	Sol–gel and spin coating	340	ITO/NiCo_2_O_4_/perovskite/PCBM/C_60_/Ag	18.02	[149]
NiCo_2_O_4_	Consuming and blade Coating	250	ITO/NiCo_2_O_4_/MAPbI_3_/PCBM/Al	15.5	[150]
MoOx	Vacuum thermal evaporation	RT	ITO/NiO_*x*_/MAPbI_3_/PCBM/BCP/Al	6.4	[151]
MoOx	thermal decomposition and spin coating	100	ITO/MoO_3_/PEDOT:PSS/MAPbI_3_/C_60_/Ag	14.87	[152]
CoOx	thermal decomposition and spin coating	300	ITO/CoO_*x*_/MAPbI_3_/PCBM/Ag	14.5	[153]
VOx	Atomic‐layer‐deposited	50	FTO/VO_*x*_/MAPbI_3_/PCBM/BCP/Ag	11.53	[154]
VOx	Hot melt quenching and spin coating	150	ITO/VO_*x*_/MAPbI_3_/PCBM/Al	14.23	[155]
WOx	Hydrothermal and spin coating	–	ITO/WO_*x*_/MAPbI_3_/PCBM/Al	7.68	[156]
TiO_2_	Hydrothermal and spin coating	500	FTO/TiO_2_/(FAPbI_3_)_1−*x*_(MAPbBr_3_)_*x*_/PTAA/Au	18.4	[157]
TiO_2_	Chemical bath deposition	–	FTO/c‐TiO_2_/MAFAPbI_3_/spiro‐OMeTAD/Au	24.8	[158]
TiO_2_	Electrolysis and electrochemical deposition	450	FTO/c‐TiO_2_/*m*‐TiO_2_/FA_0.85_MA_0.10_Cs_0.05_Pb(I_0.87_Br_0.13_)_3_/spiro‐OMeTAD/Au	20.9	[159]
TiO_2_	Precursor solution and spin coating	125	FTO/c‐TiO_2_/FA_0.9_Cs_0.1_PbI_3_/spiro‐OMeTAD/Au	18.84	[160]
TiO_2_	Pulsed laser deposition	500	FTO/c‐TiO_2_/*m*‐TiO_2_/MAPbI_3_/spiro‐OMeTAD/Ag	13.95	[161]
TiO_2_	Atomic layer deposition	100	ITO‐PEN/a‐TiO_2_/PCBM/MAPbI_3_/spiro‐OMeTAD/Au	16.74	[162]
TiO_2_	Sputtered	–	FTO/c‐TiO_2_/MAPbI_3_/spiro‐OMeTAD/Ag	17.25	[163]
TiO_2_	E‐beam evaporation	200	FTO/TiO_2_/MAPbI_3_/spiro‐OMeTAD/Au	15.39	[164]
TiO_2_	Nanoparticle slurry and spin coating	450	FTO/c‐TiO_2_/*m*‐TiO_2_/FAPbI_3_(MACl)/spiroOMeTAD/Au	24.02	[165]
SnO_2_	Chemical bath deposition	70	FTO/SnO_2_/FAPbI_3_/spiro‐OMeTAD/Au	25.8	[3]
SnO_2_	UV ozone treatment and spin coating	50	FTO/SnO_2_/Cs_0.05_(FA_0.85_MA_0.15_)_0.95_Pb(I_0.85_Br_0.15_)_3_/spiro‐OMeTAD/Au	20.5	[166]
SnO_2_	Nanoparticle slurry and spin coating	150	ITO/SnO_2_/(FAPbI_3_)_1−*x*_(MAPbBr_3_)_*x*_(PEAI)/spiro‐OMeTAD/Au	23.56	[167]
SnO_2_	Atomic layer deposition	118	FTO/SnO_2_/RbCsFAPbI_3_/spiro‐OMeTAD/Au	20.44	[168]
SnO_2_	E‐beam evaporation	180	FTO/SnO_2_/(MA_0.17_FA_0.83_)_95_Pb(I_0.83_Br_0.1_)_3_/spiro‐OMeTAD/Au	18.2	[169]
SnO_2_	Precursor solution and spin coating	180	ITO/SnO_2_/MAPbI_3_/spiro‐OMeTAD/Au	20.2	[170]
SnO_2_	Sputtered	–	FTO/SnO_2_/Cs_0.06_MA_0.27_FA_0.67_PbI_2.7_Br_0.3_/spiro‐OMeTAD/Au	20.2	[171]
SnO_2_	Plasma treatment	50/200	FTO/SnO_2_/Cs_0.05_(MA_0.17_FA_0.83_)_0.95_PbI_2.7_Br_0.3_/spiro‐OMeTAD/Au	17.8	[172]
SnO_2_	Combustion procedure and spin coating	200	FTO/SnO_2_/Cs_0.05_(MA_0.17_FA_0.83_)_0.95_Pb(I_0.83_Br_0.17_)_3_/spiro‐OMeTAD/Au	19.12	[173]
SnO_2_	Thermal evaporation	500	FTO/SnO_2_/MAPbI_3_/spiro‐OMeTAD/Au	16.79	[174]
SnO_2_	Sol–gel method and spin coating	180	FTO/SnO_2_/(MA/FA/Cs) Pb(I/Br)_3_/spiro‐OMeTAD/Au	20.3	[175]
SnO_2_	Microwave synthesized	130	ITO/SnO_2_/MAPbI_3_/spiro‐OMeTAD/Au	14.2	[176]
SnO_2_	Photonically annealed and spin coating	–	FTO/SnO_2_/MAPbI_3_/PTAA/Au	15.3	[177]
SnO_2_	Spray pyrolysis	450	AZO/c‐SnO_2_/mp‐SnO_2_/MAPbI_3_/spiro‐OMeTAD/Au	13.1	[178]
SnO_2_	Template etching	60	FTO/SnO_2_/MAPbI_3_/P3HT/Au	12.1	[179]
ZnO	Sputtered	–	ITO/ZnO/MAPbI_3_/spiro‐OMeTAD/Au	15.9	[180]
ZnO	Atomic layer deposition	150	ITO/ZnO(TiO_2_)/MA_0.1_FA_0.75_Cs_0.15_PbI_2.9_Br_0.1_/spiro‐OMeTAD/Ag	18.26	[181]
ZnO	Chemical vapor deposition	500	FTO/ZnO/MAPbI_3_/spiro‐OMeTAD/Ag	11.75	[182]
ZnO	Hydrothermal and spin coating	350	FTO/c‐ZnO/ZnO/MAPbI_3_/spiro‐OMeTAD/Au	15.92	[183]
ZnO	Combustion and spin coating	200	ITO/ZnO/Cs_0.05_FA_0.81_MA_0.14_PbI_2.55_Br_0.45_/spiro‐OMeTAD/Au	19.84	[184]
Zn_2_SO_4_	Reflux condensation and spin coating	500	FTO/Zn_2_SO_4_/Cs_0.05_(MA_0.17_FA_0.83_)_0.95_Pb(I_0.83_Br_0.17_)_3_/spiro‐OMeTAD/Au	20.1	[185]
Zn_2_SO_4_	Hydrothermal and spin coating	450	FTO/c‐TiO_2_/mp‐Zn_2_SO_4_/MAPbI_3_/spiro‐OMeTAD/Au	17.21	[186]
Zn_2_SO_4_	Chemical bath deposition	450	FTO/Zn_2_SO_4_/Cs_0.05_(MA_0.15_FA_0.85_)_0.95_Pb(Br_0.15_I_0.85_)_3_/spiro‐OMeTAD/Au	21.3	[187]
BaSnO_3_	Coprecipitation and spin coating	500	FTO/c‐TiO_2_/SnO_2_/mp‐BaSnO_3_/(FAPbI_3_)_0.93_(MAPbBr_3_)_0.07_/spiro‐OMeTAD/Au	21.3	[188]
ZnTiO_3_	Solution and spin coating	150	ITO/ZnTiO_3_/Cs_0.05_FA_0.81_MA_0.14_PbI_2.55_Br_0.45_/spiro‐OMeTAD/Au	19.8	[189]
SrTiO_3_	Co‐precipitation and spin coating	150	ITO/SrTiO_3_/C_60_/Cs_0.07_FA_0.73_MA_0.2_0PbI_2.53_Br_0.47_/spiro‐OMeTAD/Au	19	[190]
WO_3−*X*_	E‐beam evaporation	300	FTO/WO_3−*X*_/MAPbI_3_/spiro‐OMeTAD/Au	10.3	[191]
Sn_2_O_3_	Nanoparticle slurry and spin coating	140	FTO/Sn_2_O_3_/Cs_0.05_MA_0.15_FA_0.85_Pb_1.05_I_3.15_/spiro‐OMeTAD/Ag	22.36	[27]
Sn_3_O_4_	Nanoparticle slurry and spin coating	140	FTO/Sn_3_O_4_/Cs_0.05_MA_0.15_FA_0.85_Pb_1.05_I_3.15_/spiro‐OMeTAD/Ag	21.83	[27]

**Figure 2:**
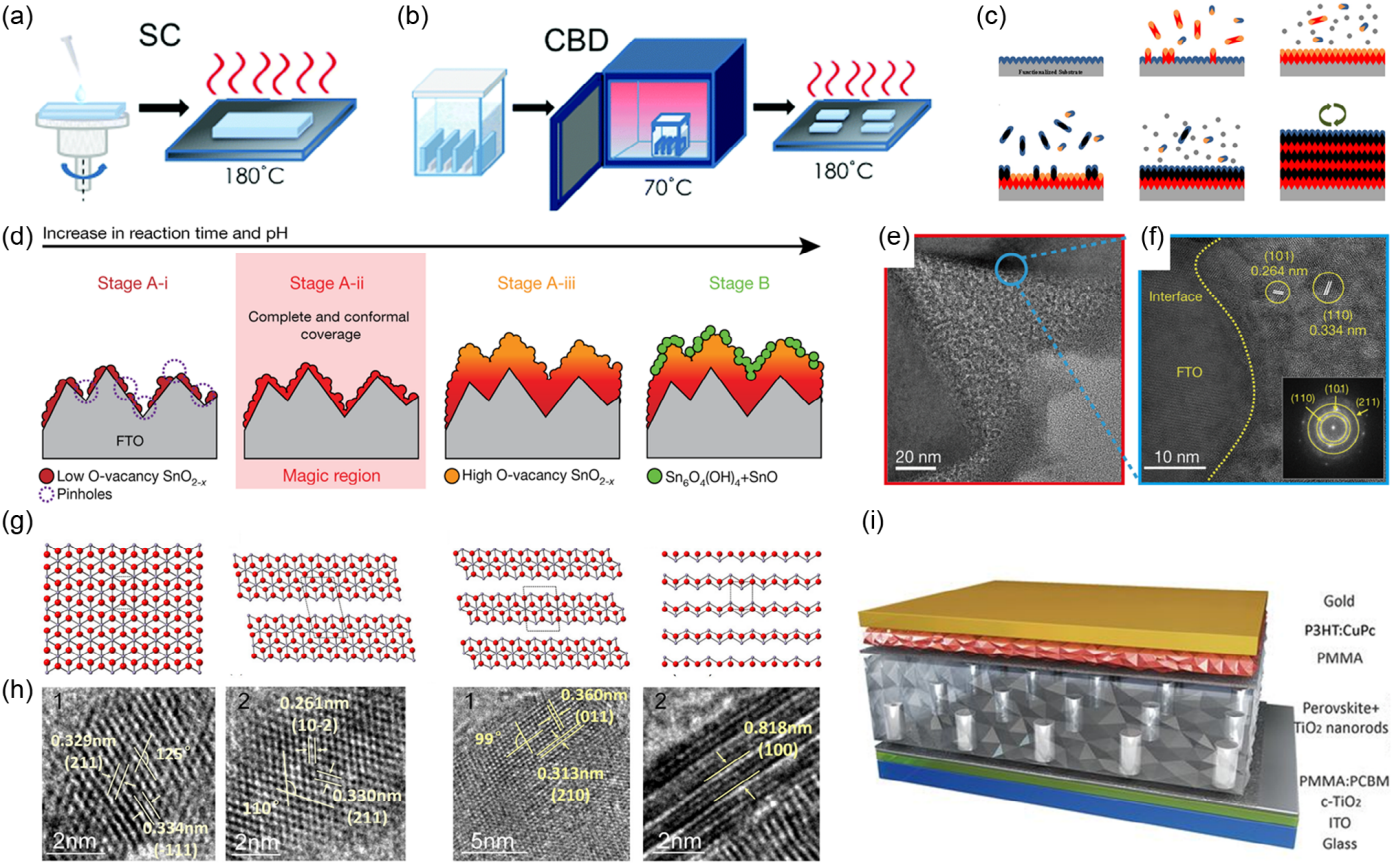
a) Spin coating (SC) of CTLs. b) Chemical bath deposition (CBD) of CTLs. a,b) Reproduced with permission.^[^ ^13^ ^]^ Copyright 2016, Royal Society of Chemistry. c) Schematic of atomic layer deposition (ALD) for CTLs. Reproduced with permission.^[^ ^14^ ^]^ Copyright 2014, Elsevier. d) Schematic illustration of the progress of the reaction highlighting stage A‐ii as the magic region, which exhibits ideal film coverage, morphology, and chemical composition. e) High‐resolution TEM images of films prepared up to stage A‐ii, the inset in (f) shows a fast Fourier transform pattern of the TEM image. d–f) Reproduced with permission.^[^ ^15^ ^]^ Copyright 2021, Springer Nature. g) Theoretical crystal structure of SnO_2_, Sn_2_O_3_, Sn_3_O_4_ and SnO (from left to right). h) HRTEM images of Sn_2_O_3_ (left) and Sn_3_O_4_ (right) particles. g,h) Reproduced with permission.^[^ ^27^ ^]^ Copyright 2020, American Chemical Society. i) Schematic of nanopattern perovskite cell. Reproduced with permission.^[^ ^30^ ^]^ Copyright 2021, American Association for the Advancement of Science.

The properties of CTLs are not only affected by their components and crystal structure but also depend on processing details. Different preparation processes can induce changes in defects, morphology, surface groups, energy band structure, and electrical properties, all of which will affect the performance of CTLs and related PSCs. The regulation of preparation methods and processes can significantly impact the semiconductor properties and film coverage. Elham et al. prepared SnO_2_ thin films using ALD, spin‐coating (SC), and spin‐coating‐chemical bath deposition (SC‐CBD), respectively (**Figure** **2**a–c).^[^ ^13^, ^14^ ^]^ PSCs based on these three ETLs obtained a PCE of 19.0%, 19.5%, and 20.8%, respectively. They concluded that the surface of ETL prepared by ALD method was too smooth, which might have poor contact with perovskite, resulting in increased series resistance. Although the roughness of the ETL prepared by SC increased, it also produced more surface defects and limited performance improvement. The SC‐CBD technique was found to effectively reduce surface defects, thereby suppressing nonradiative recombination and leading to improved device performance. Given these advantages, the CBD was a widely adopted method for fabricating dense SnO_*x*_ ETLs with excellent coverage, appropriate thickness, and minimal defects (Figure 2d–f).^[^ ^15^ ^]^ The pH of the solution was reported to have a critical effect on the water bath synthesis of SnO_*x*_. The increasing pH and reaction time resulted in greater coverage of the SnO_*x*_ film and increased oxygen vacancies, as well as the formation of additional products, SnO and Sn_6_O_4_(OH)_4_. By modulating the CBD process, the PSC based on SnO_*x*_ ETL achieved a certified efficiency of 25.2% and exhibited no significant degradation in efficiency after 3600 h storage.

For indirect deposition, the nanoparticle morphology has a significant impact on the performance of CTLs. One‐dimensional ETL nanomaterials such as nanorods (NRs), nanowires (NWs), and nanotubes have been applied to the fabrication of nanostructured ETLs which have an open structure for effective filling of perovskite and directional charge transfer.^[^ ^16^, ^17^, ^18^ ^]^ Meanwhile, they have high specific surface area and strong contact with perovskite, which could greatly facilitate charge injection and dramatically reduce charge accumulation and recombination at the ETL/perovskite interface.^[^ ^19^, ^20^, ^21^, ^22^ ^]^

Two‐dimensional nanosheets are characterized by their high coverage and well‐ordered arrangement, which promote better contact between the perovskite and the nanosheet, and increase the charge collection efficiency. In addition, the compact nanosheet layer serves to isolate external moisture, making the device more stable. Further precise regulation of nanostructure morphology and optimization of interfacial contact provide a potential solution for achieving breakthroughs in device performance.^[^ ^23^, ^24^, ^25^, ^26^ ^]^ Mixed‐valence tin oxide Sn_2_O_3_ and Sn_3_O_4_ were also reported as ETLs for PSCs.^[^ ^27^ ^]^ The van der Waals crystal structure made Sn_3_O_4_ tends to grow into nanosheets. Sn_2_O_3_ and Sn_3_O_4_ demonstrated good wettability with perovskite and exhibited good UV stability (Figure 2g,h).

By employing the solvothermal method to produce well‐separated NWs with a controllable length‐to‐diameter ratio, the electron transport rate was found to be 200 times faster compared to that of mesoporous films.^[^ ^28^ ^]^ Meanwhile, the morphology, length, width, and porosity of NRs and NWs were further regulated to facilitate carrier extraction and directional transport. TiO_2_ NRs with adjustable lengths of 70–200 nm were synthesized by the solvothermal method.^[^ ^29^ ^]^ The NRs with a length of 110 nm had large pore size, good interfacial contact, and efficient carrier transfer. To improve the interfacial contact, further, passivate surface defects of ETL and regulate crystallization, the dual treatment was used to pattern TiO_2_ NRs. As a result, the device achieved an efficiency of 21.6% and a fill factor of 84% over an effective area of 1 cm^2^, while simultaneously suppressing interfacial charge recombination and ensuring low interfacial resistance loss, fast carrier extraction, and transport (Figure 2i).^[^ ^30^ ^]^

The morphology modulation of MO HTLs has been less explored than that of ETLs, although similar regulatory mechanisms were involved. Li et al. reported a simple hydrothermal method to synthesize nanosheets, NWs, and mixed mesoporous structures of NiCo_2_O_4_. Compared to nanoparticles, NiCo_2_O_4_ NWs avoided agglomeration, and exhibited excellent conductivity and hole mobility, which suppressed interfacial recombination and accelerated charge extraction.^[^ ^31^ ^]^ Meanwhile, the nanosheets of NiO_*x*_ with their high porosity and large specific surface area facilitated the diffusion and permeation of perovskite. These properties led to good interfacial contact, fast carrier extraction and transport, and reduced hysteresis effect of PSCs.^[^ ^32^, ^33^ ^]^

## 3 Interface Regulation of MO‐CTLs

Highly efficient and stable PSCs rely on precise regulation of interfaces. The presence of defect states at the CTL surfaces could lead to significant interface recombination, with the defect density being two orders of magnitude higher than that of the bulk phase. The surface properties of CTLs also affect the energy level alignment, mechanical contact, and wettability. Meanwhile, a stable PSC requires a tough interface against the stimulation of light, heat, and electric field. Otherwise, undesired reactions and migrations would occur at the interface. In this section, we summarize various interfacial modification strategies for improving the PCE and stability of PSCs.

### 3.1 Interface Modification of ETL

#### 3.1.1 Inorganic Interface Layer

**Figure 3:**
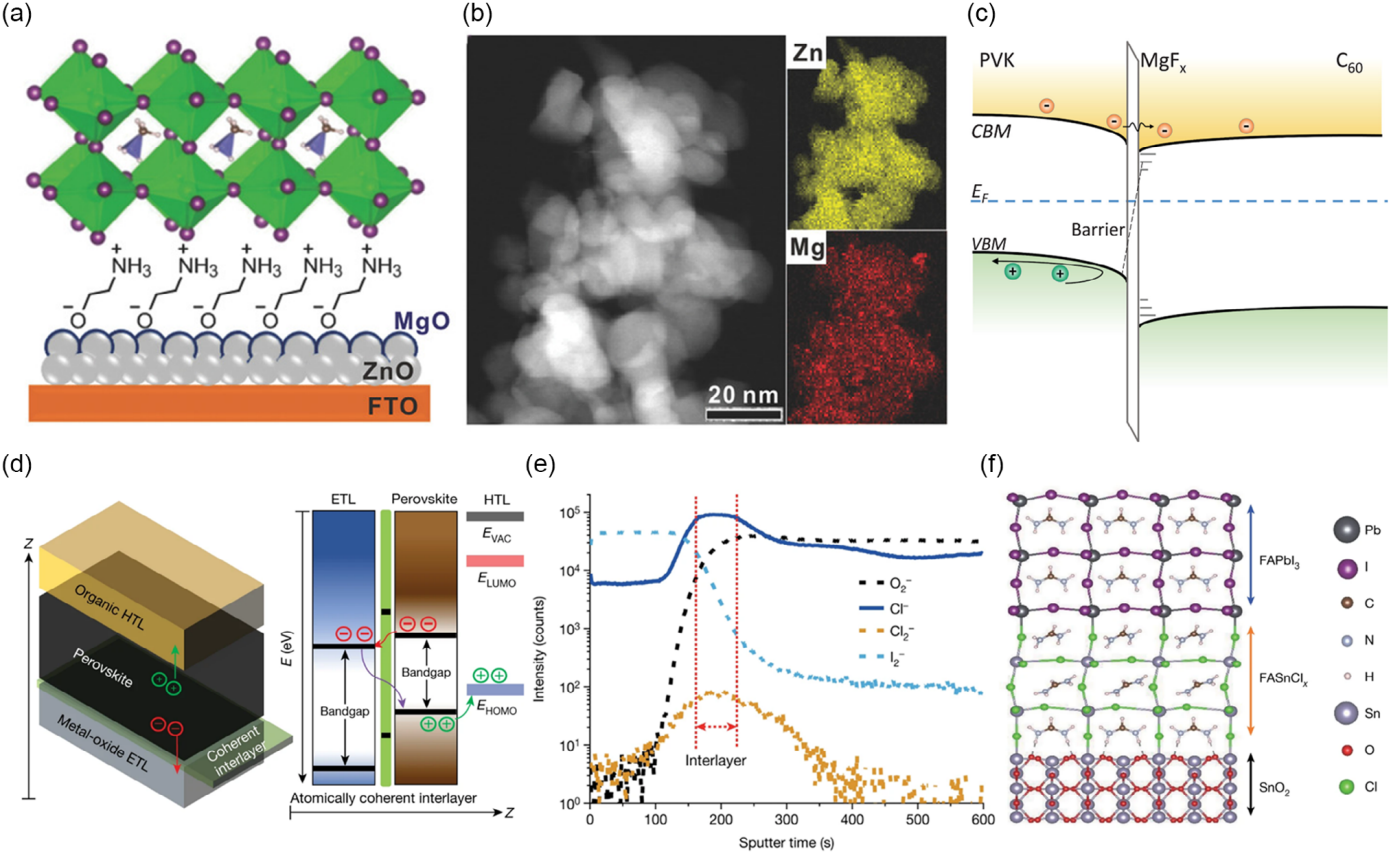
a) Schematic illustration of a planar PSC device modified with ZnO‐MgO‐EA^+^. b) HAADF‐STEM images and elemental maps of ZnO‐MgO‐EA^+^ sample. a,b) Reproduced with permission.^[^ ^41^ ^]^ Copyright 2018, Wiley‐VCH. c) Energy level diagram of the perovskite/C_60_ interface with MgF_*x*_ insertion layer. Reproduced with permission.^[^ ^43^ ^]^ Copyright 2022, American Association for the Advancement of Science. d) Layered stack of the ETL, perovskite and HTL, with an interlayer and energy diagrams of the ETL, perovskite, and HTL. e) ToF‐SIMS depth profiles for the perovskite on FTO. f) Simulation of the formation of the interlayer between perovskite and SnO_2_. d–f) Reproduced with permission.^[^ ^3^ ^]^ Copyright 2021, Springer Nature.

To modify the ETL/perovskite interface and regulate the electrical properties of ETLs, inorganic materials have been utilized as overlay materials due to their good stability and special electrical properties. MgO, MgF_2_, SrO, Al_2_O_3_, ZrO_2_, SiO_2_, and CaCO_3_ have been considered as insulating layers to suppress charge recombination at the ETL/perovskite interface and facilitate defect passivation, band alignment, and dipole formation.^[^ ^34^, ^35^, ^36^, ^37^ ^]^ Due to the electron tunneling effect, the interface layer allows one type of carriers to pass through while blocking the other.^[^ ^38^ ^]^ Wang et al. found that the interfacial layer of MgO suppressed the formation of δ‐phase perovskite, reduced the work function of ETL, and decreased interfacial charge recombination. As a result, the hysteresis effect of the device was substantially reduced and the stability of the device was enhanced.^[^ ^39^ ^]^ For the ZnO ETL, the organic cations of perovskite were deprotonated due to surface groups and alkalinity of ZnO, resulting in the perovskite decomposition.^[^ ^40^ ^]^ The introduction of an interfacial layer was particularly important for both performance and stability improvements of PSCs based on ZnO. Cao et al. coated the ZnO layer with a thin film of MgO and protonated ethanolamine (EA) to improve interfacial compatibility and enhance charge transport. As a result, they achieved the best efficiency of 21.1% with no hysteresis in their PSCs (**Figure** **3**a,b).^[^ ^41^ ^]^

Besides the interface between ETL and perovskite, these inorganic oxides could also serve as a dense barrier layer at the interface between electrodes and ETL. The use of the MgO nanolayer with a lower VBM has been found to be effective in hole blocking.^[^ ^42^ ^]^ In addition, MgF_*x*_ and LiF were also commonly utilized as the interfacial layer of PSCs to isolate water and oxygen and reduced ion migration for improved device stability (Figure 3c).^[^ ^43^, ^44^, ^45^ ^]^

Treating TiO_2_ with TiCl_4_ resulted in a smoother interface layer that covered the conductive electrode more effectively, thereby improving the contact at the TiO_2_/perovskite interface, promoting the carrier extraction and inhibiting the charge recombination.^[^ ^46^, ^47^ ^]^ In addition, it was worth noting that the treatment of fine chlorination on the surface also had the potential to introduce coherent layers. The additional coherent layer formed by the bonding of Cl at the SnO_2_/perovskite interface eliminated the effect of interfacial defects and increased the efficiency of the device to 25.8% (certified 25.5%) (Figure 3d–f).^[^ ^3^ ^]^

#### 3.1.2 Interface Salt Treatment

Besides the previously mentioned inorganic overlays, treating CTLs with salts such as CsBr, CsCl, NaCl, K_2_CO_3_, Sb_2_S_3,_ and KI also led to enhanced device performance.^[^ ^48^, ^49^, ^50^, ^51^, ^52^, ^53^ ^]^ Such treatment improved the crystallization of perovskite and passivated interface defects. The CsBr modification reduced the work function of TiO_2_ and the areal density of pinholes. The Cs^+^ could effectively suppress nonradiative recombination at the interface.^[^ ^54^ ^]^ Additionally, treating TiO_2_ ETL with CsCl enhanced the electron injection and thermal stability of PSCs.^[^ ^55^ ^]^

**Figure 4:**
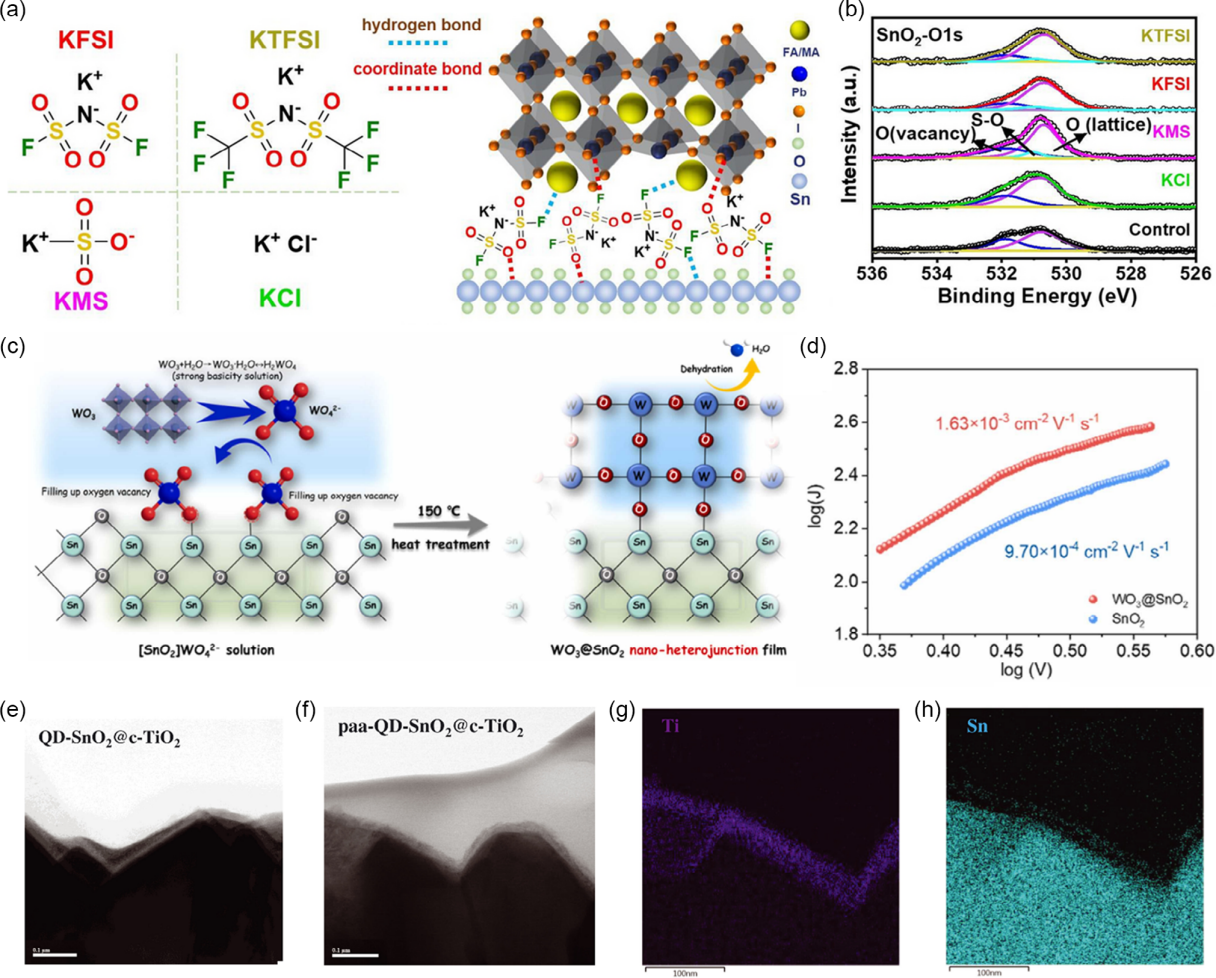
a) Molecular structures of potassium salts used for modifying buried interface and schematic illustration of the interaction of modifiers and functional layers. b) O 1s XPS spectra of the control and modified SnO_2_ layers. a,b) Reproduced with permission.^[^ ^57^ ^]^ Copyright 2022, Springer Nature. c) Schematic diagram of the transformation process from solution to film with WO_3_ added to alkaline SnO_2_ colloidal solution. d) Electron mobility calculation using the SCLC model with the device structure of FTO/ETL/PCBM/Au. c,d) Reproduced with permission.^[^ ^72^ ^]^ Copyright 2022, Elsevier. The cross‐sectional TEM images of QD‐SnO_2_@c‐TiO_2_ (e) and paa‐QD‐SnO_2_@c‐TiO_2_ (f) over the FTO substrates. EDS elemental analysis of Ti (g) and Sn (h) for paa‐QD‐SnO_2_@c‐TiO_2_. e–h) Reproduced with permission.^[^ ^68^ ^]^ Copyright 2022, American Association for the Advancement of Science.

In contrast, the dual passivation effect of alkali metal salts such as LiCl, NaCl, KCl, RbCl, RbF, and Na_2_S at the perovskite and ETL interfaces to enhance device performance was also widely reported.^[^ ^48^, ^56^ ^]^ The anions were effective in passivating defects such as oxygen vacancies on the ETL surface due to their electronegativity and Lewis acid‐base coordination, while the cations reduced defect states in the perovskite layer. Chen et al. fixed the cation as K^+^, obtained improved synergy between fluorine and sulfonyl functional groups through the functional group design of various anions such as Cl^−^, MS^−^, TFSI^−^, and FSI^−^.^[^ ^57^ ^]^ By substantially passivating interfacial defects and oxygen vacancies on the surface of ETL, the device based on the SnO_2_ ETL with KFSI achieved an efficiency of 24.17% (**Figure** **4**a,b). Zhuang et al. adopted a dual passivation strategy to act on the ETL/perovskite interface with RbF.^[^ ^58^ ^]^ The bond of F to Sn led to a bias in the electron cloud density, which increased the electron mobility of SnO_2_. Rb^+^ actively migrated into the lattice interstices of perovskite, inhibiting ion migration and reducing nonradiative recombination. The treatment of SnO_2_ with EuCl_3_ was considered to have a dual passivation effect due to the presence of Eu^3+^ ions.^[^ ^59^ ^]^ Moreover, the enrichment of Eu^3+^ ions at the interface also prevented the perovskite decomposition caused by water penetration, thus improving the device stability.

#### 3.1.3 Double‐Layer ETL

The insufficient charge extraction capability of the ETL interface and the imperfect interfacial energy band alignment were two critical factors limiting the efficiency of PSCs. However, compared to the single ETL, the double‐layer stacked ETL was better equipped to satisfy these two requirements.^[^ ^60^, ^61^, ^62^, ^63^ ^]^ For example, SnO_2_/BaSnO_3_,^[^ ^64^ ^]^ ZnS/ZnO,^[^ ^65^ ^]^ In_2_O_3_/TiO_2_, SnO_2_/ZnO,^[^ ^66^, ^67^ ^]^ SnO_2_/TiO_2_,^[^ ^68^, ^69^ ^]^ BaTiO_3_/TiO_2_,^[^ ^70^ ^]^ and In_2_O_3_/SnO_2_^[^ ^71^ ^]^ were reported to be used as double‐layer ETL. The utilization of double‐stacked ETL reduced the roughness and pinholes in ETL films, thereby reducing the charge recombination. Li et al. prepared the SnO_2_/WO_3_ double layer as the ETL by in situ peptization using WO_4_^−^ in an aqueous solution of SnO_2_, maximizing passivation of surface hydroxyl groups and oxygen vacancies (Figure 4c,d).^[^ ^72^ ^]^ At the same time, the formed heterojunction of SnO_2_ and WO_3_ facilitated charge transport. As a result, the optimized device delivered an efficiency of 23.6% and an ultrahigh FF of 85.8%. Another double‐layer ETL, TiO_2_/SnO_2_, was constructed to improve carrier extraction and suppress nonradiative recombination in PSCs. The device achieved a certified efficiency of 25.4%, indicating the potential of double‐layer stacked ETLs for improving the performance of PSCs (Figure 4e–h).^[^ ^68^ ^]^

#### 3.1.4 Self‐Assembled Interface Monolayer

**Figure 5:**
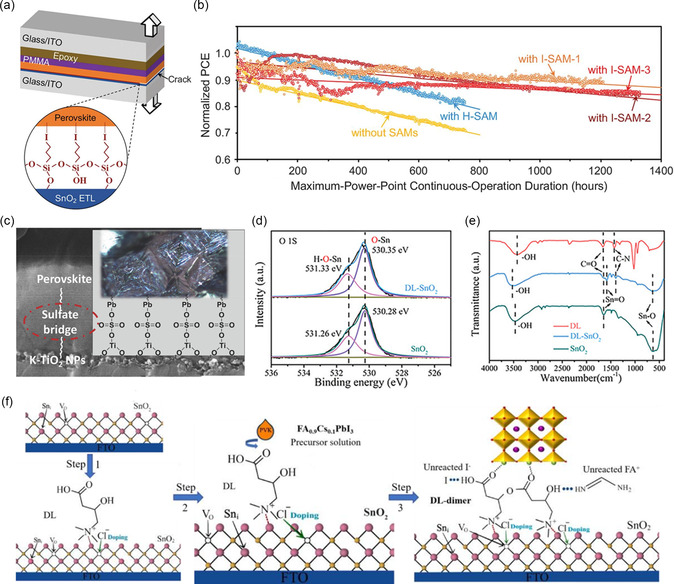
a) Schematic illustration of the sandwich DCB specimen for toughness testing (not to scale). PMMA, poly (methyl methacrylate). b) Operational stability of PSCs. a,b) Reproduced with permission.^[^ ^76^ ^]^ Copyright 2021, American Association for the Advancement of Science. c) Schematic illustration of doped‐TiO_2_ mesoporous layer and the surface states bonding with ETL and perovskite. Reproduced with permission.^[^ ^77^ ^]^ Copyright 2018, Wiley‐VCH. d) The XPS spectra of O 1s for SnO_2_ and DL‐SnO_2_ film. e) FTIR spectra of DL, DL‐SnO_2_, and SnO_2_ films. f) Proposed working mechanism of DL at perovskite/SnO_2_ interface. d–f) Reproduced with permission.^[^ ^78^ ^]^ Copyright 2023, Wiley‐VCH.

The functional self‐assembled monolayer (SAM), such as C_60_‐SAM, silane SAM and other SAM system could be used to modify the interface of ETL/perovskite. The formation of stable electron coupling on the surface of the ETL could allow for adjustment of its characteristics, such as surface defect passivation and enhancement of interface charge transfer.^[^ ^73^ ^]^ The SAM could also affect the energy level by imparting a dipole moment at the interface.^[^ ^74^ ^]^ In addition, SAM had a significant enhancement of the interfacial stability of the device and could be used to resist bending in flexible devices.^[^ ^75^ ^]^ Dai et al. reported the use of iodine‐terminated self‐assembled monolayer (I‐SAM) in PSCs significantly increased the bond toughness and stability at the interface by reacting with surface hydroxyl groups.^[^ ^76^ ^]^ The device efficiency processed with I‐SAM was increased from 20.2% to 21.4% and had a lower hysteresis effect. The device kept 80% of its initial PCE after aging under continuous maximum power point tracking for 4,000 h (**Figure** **5**a,b). The KTFSI, LiTFSI, CsTFSI, and NaTFSI were also used to treat mp‐TiO_2_ and perovskite interfaces. Due to the chemical affinity of lead to sulfate, the anion bridged the ETL and lead‐based perovskite, and improved device performance (Figure 5c).^[^ ^77^ ^]^ Lu et al. treated the interface between SnO_2_ and perovskite via DL‐carnitine hydrochloride. The cation formed an interface layer by interacting with SnO_2_ through electrostatic coupling and interacting perovskite via the hydrogen bonds. This treatment led to a record PCE value of 25.24% for FACsPbI_3_‐based PSCs (Figure 5d–f).^[^ ^78^ ^]^

### 3.2 Interface Modification for HTL

Modifications such as oxide interlayers, salt interlayers, and organic layers could also be applied to MO‐HTLs.^[^ ^79^, ^80^ ^]^ The addition of both anions and cations in Na_2_S demonstrated a synergistic effect that improved the interfacial contact between NiO_*x*_ and perovskite. This improvement enhanced the crystallinity of perovskite and the conductivity of NiO_*x*_, while reducing the nonradiative recombination at the interface and significantly boosting the device performance.^[^ ^81^ ^]^ Similarly, amphiphilic molecules, SAMs, and long‐chain alkylamines were used to inhibit the deprotonation reaction of NiO_*x*_ for perovskite and passivated charged defects such as Ni^3+^ on the surface through Lewis acid‐base coordination.^[^ ^80^, ^82^, ^83^, ^84^ ^]^ The surface modification treatment of NiO_*x*_ by Triton X100 improved the energy level matching, accelerated the carrier extraction and transport, while the efficiency and stability of the device were dramatically enhanced due to the covering layer formed by hydrophobic Triton X100.^[^ ^82^ ^]^ The device achieved a high efficiency of 22.35% and maintained 88.4% of the initial efficiency after being stored in air for 1070 h. The multifunctional additive 3‐hydrazinylbenzoic acid was used to inhibit the deprotonation reaction of organic cations and the oxidation of *I* ^−^, with the –NHNH_2_ and –COOH groups acting synergistically. This approach effectively passivated interfacial defects, leading to an increase in carrier lifetime and suppression of nonradiative recombination. The device achieved a PCE of 23.3% with excellent operational stability.^[^ ^84^ ^]^ The carbazole‐based SAM such as Me‐4PACz was demonstrated very effective for constructing the hole selective contact and now widely applied for efficient inverted PSCs.

## 4 Element Doping for MO‐CTL

It is reported that the actual electron mobility of conventional ETLs is much lower than that of the conventionally used HTLs (PTAA, spiro‐OMeTAD, etc.). Some MO‐HTLs also have relatively low hole mobility. The imbalance of electron and hole fluxes at the interface could lead to charge accumulation at the interface, which further affects the device performance.^[^ ^85^, ^86^ ^]^ Doping is a promising method for adjusting the conductivity and carrier mobility. Meanwhile, doping can also minimize and even alleviate the lattice distortion, which reduces the original carrier migration barrier. In principle, the doping of MOs requires consideration of both the metal element and the oxygen element.^[^ ^87^, ^88^, ^89^ ^]^ Relying solely on the valence state of the doped metal to judge the doping type and carrier concentration is not comprehensive enough. The introduced dopant ions generally exist in the state of substitution and interstitial lattice occupation.^[^ ^35^, ^90^ ^]^ In addition, some ions may enter the lattice gap to provide additional carriers. Furthermore, the doping of ions is extremely susceptible to affect the formation energy of oxygen vacancies which could influence the self‐doping effect of the MO, bringing about changes in semiconductor properties.^[^ ^91^ ^]^ The modulation of the self‐doping effect also enables the precise modulation of electrical properties. Better energy band matching can also be achieved by doping, which accelerates carrier injection and transport, and reduces interfacial charge recombination losses. The CBM and VBM of TiO_2_ are dominated by Ti 3 d and O 2p orbitals, respectively, and those for SnO_2_ are Sn 5s and O 2p.^[^ ^92^ ^]^ It is generally accepted that O p orbitals are relatively localized and fixed.^[^ ^93^ ^]^ As a result, metal element doping can directly affect the position of the CBM.

### 4.1 Element Doping for ETL

Alkali and alkaline earth metals are favored for doping ETLs due to their lower electronegativity and active chemical properties. The Li doping strategy induced a partial reduction of Ti^4+^ to Ti^3+^ within the TiO_2_ lattice and passivated electronic defect, resulting in faster charge transfer.^[^ ^94^, ^95^, ^96^ ^]^ The introduction of Li was able to significantly modulate the energy band alignment, enhance the conductivity and carrier mobility of TiO_2_, accelerate the carrier extraction and transport at the interface, and suppress nonradiative recombination.^[^ ^97^ ^]^ Similarly, the doping of Li was reported to enhance the conductivity of SnO_2_ and ZnO, and affect the energy band structure.

**Figure 6:**
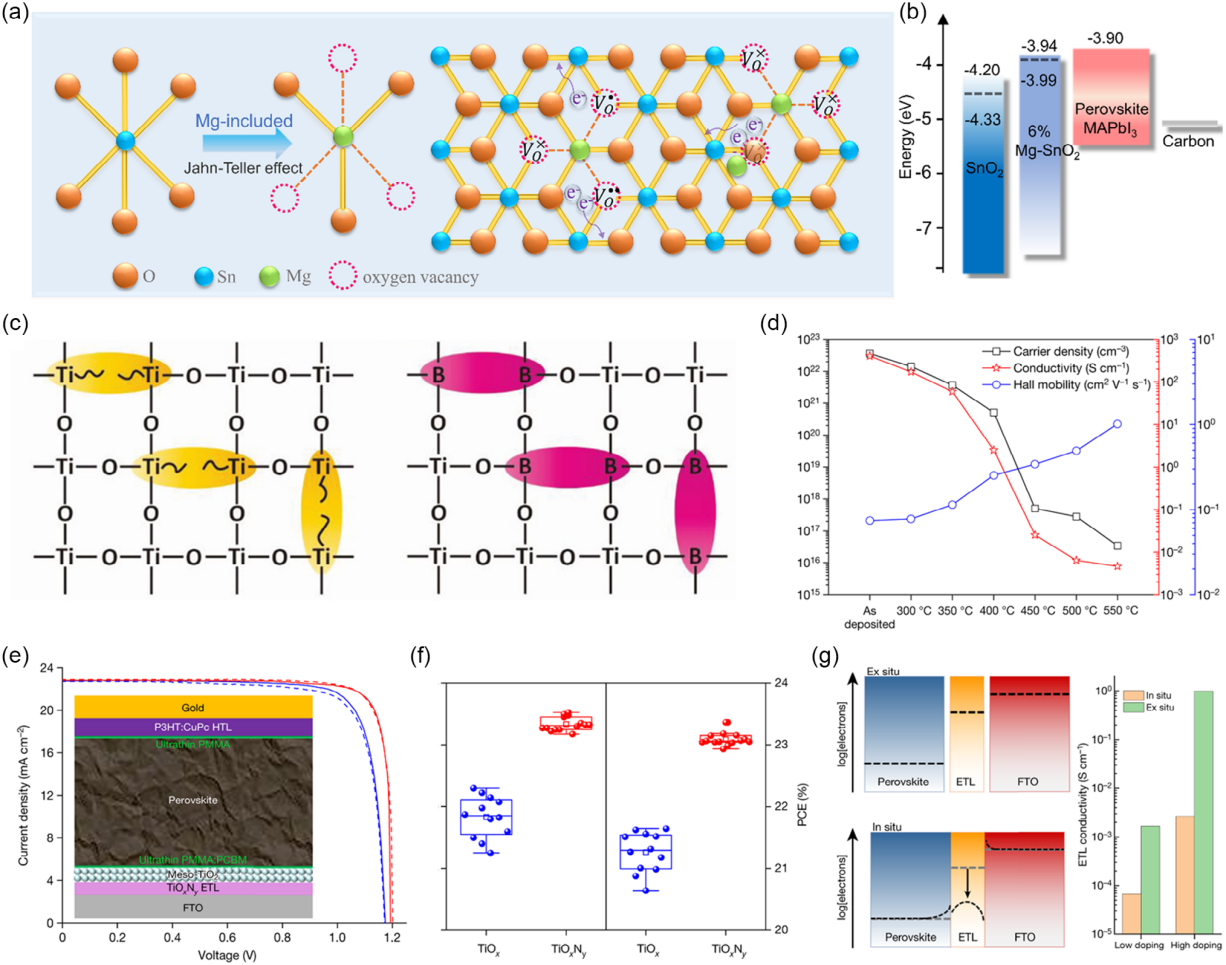
a) Diagrammatic sketch for self‐doping induced by the synergy of Mg_Sn_ and Mg_i_ in 6% Mg‐SnO_2_. b) Energy level alignment between 0% or 6% Mg‐SnO_2_ and halide perovskites. a,b) Reproduced with permission.^[^ ^35^ ^]^ Copyright 2022, Wiley‐VCH. c) Boron substitution in the Ti sites passivates these defects. Reproduced with permission.^[^ ^101^ ^]^ Copyright 2019, Wiley‐VCH. d) Carrier density, conductivity, and Hall mobility of TiO_*x*_N_*y*_ films, e) *J–V* curves of the champion 1 cm^2^ TiO_*x*_‐based cell. f) FF and PCE distribution for the TiO_*x*_‐based cells (12 cells) and the TiO_*x*_N_*y*_‐based cells (14 cells). g) Schematic representation and diagram of the ex situ and in situ conductivity of two doping levels of the ETL. d–g) Reproduced with permission.^[^ ^4^ ^]^ Copyright 2022, Springer Nature.

Among divalent metal ions, Mg and Sr are the most commonly used. The doping of SnO_2_ with Mg salt was found to generate oxygen vacancies after occupying the lattice Sn sites. The occupation of lattice gaps by Mg ions induced the ionization of oxygen vacancies, providing additional carriers and simultaneously passivating the defects. Mg doping also improved the energy level matching between SnO_2_ and perovskite, and the formation of MgO suppressed interfacial recombination, significantly improving the device performance (**Figure** **6a** **,**b).^[^ ^35^ ^]^ In the ZnO ETL, the doping of Mg was reported to improve the surface properties, inhibit the occurrence of deprotonation reactions, and significantly improve the device stability.^[^ ^98^ ^]^ Sr was reported to be used in the doping of ETLs. For BaSnO_3_, the CBM and VBM were mainly composed of Sn 5s/5p and O 2p orbitals. The substitution of Sr led to a shortening of the average bond length of Sn–O, which resulted in an enhancement of the hybridization strength and an increased splitting of the crystal field, leading to an upward shift of the CBM position. As a result, the device efficiency based on Sr_0.2_Ba_0.8_SnO_3_ ETL was improved to 21%.^[^ ^99^ ^]^

Al and B are the more typical main group elements for trivalent doping. It was believed that the doping of trivalent metal elements was thought to replace Ti^3+^ in TiO_2_, thus forming a more stable bond with the surrounding oxygen and passivating the defects. In Al‐doped ZnO, Al occupied both the lattice Zn sites and the lattice interstitials, which provided carriers and enhanced the conductivity of ZnO.^[^ ^100^ ^]^ The sol–gel‐processed boron‐doped TiO_2_ (B‐TiO_2_) was considered to reduce hysteresis effect and obtain better PCE of the device.^[^ ^101^ ^]^ The B doping was able to passivate defects and reduce oxygen vacancies, resulting in more efficient charge extraction, and increased the electron mobility of TiO_2_ from 3.30 × 10^−5^ to 1.69 × 10^−4^ cm^2^ V^−1^ s^−1^ (Figure 6c). The doping of Al and In was also significant for the enhancement of carrier mobility and conductivity of SnO_2_ and TiO_2_.^[^ ^102^, ^103^ ^]^

In addition, Sn,^[^ ^104^, ^105^ ^]^ N, Cs,^[^ ^106^ ^]^ Cl,^[^ ^107^ ^]^ F,^[^ ^69^, ^108^ ^]^ and other main group elements were used in doping ETL to regulate the electrical conductivity, defect states, and energy band structure of ETL as well. Furthermore, partial doping could also improve the contact interface between perovskite and ETL, resulting in larger average grain size and better crystallinity of perovskite. By delicately controlling the annealing atmosphere and temperature, TiO_*x*_N_*y*_ films with suitable energy level alignment, carrier concentration, conductivity, mobility, and transmission were obtained.^[^ ^4^ ^]^ The optimized ETL exhibited excellent charge transport properties, resulting in a certified PCE of 22.6% for 1 cm^2^ PSCs. Simulations showed that the high doping of ETL (⪆10^17^ cm^−3^) prevented electron depletion in the positive space charge region of both perovskite/ETL and FTO/ETL heterojunctions and which was necessary for PSCs to achieve FFs in excess of 85% (Figure 6d–g).

**Figure 7:**
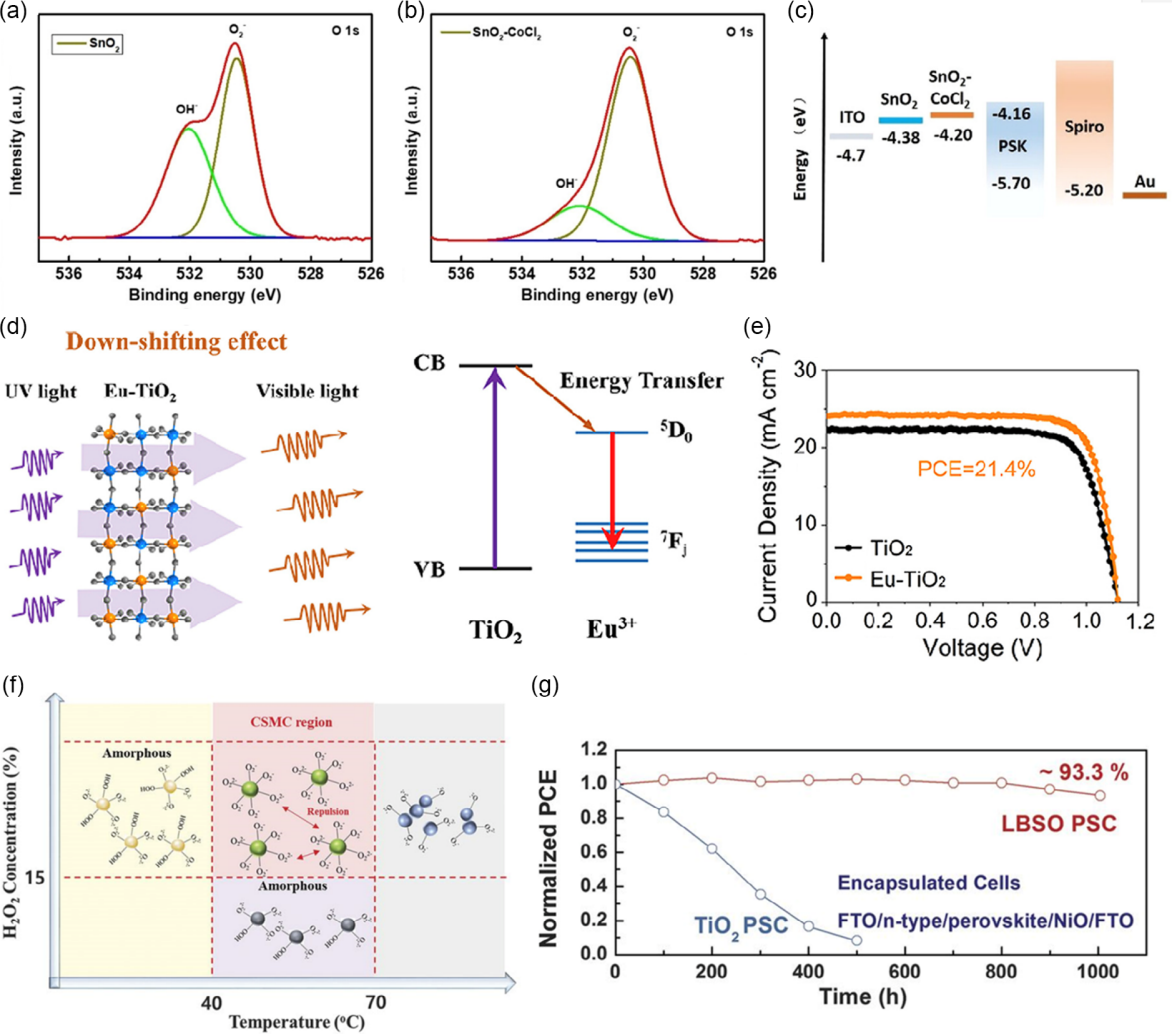
a) XPS of O 1s for pristine SnO_2_ and b) SnO_2_‐CoCl_2_. c) High‐resolution TEM images of pristine SnO_2_ and SnO_2_‐CoCl_2_. a–c) Reproduced with permission.^[^ ^120^ ^]^ Copyright 2021, American Chemical Society. d) Illustrated mechanism of down‐shifting transition from UV photons to visible luminescence. e) *J–V* curves of the champion TiO_2_‐based cells. d–e) Reproduced with permission.^[^ ^123^ ^]^ Copyright 2020, Elsevier. f) Schematic illustration of the formation map for the superoxide precursor colloidal solution. g) Long‐term photostability test under constant AM 1.5 G illumination with a metal‐halide lamp, including UV radiation for two encapsulated devices. f,g) Reproduced with permission.^[^ ^125^ ^]^ Copyright 2017, American Association for the Advancement of Science.

Among the transition elements, the typical ETL doping elements are Zn,^[^ ^109^ ^]^ Co, Zr,^[^ ^110^, ^111^ ^]^ Y,^[^ ^112^, ^113^, ^114^ ^]^ Nd,^[^ ^115^, ^116^ ^]^ W,^[^ ^117^ ^]^ and Ta.^[^ ^118^ ^]^ These elements typically have only one or two electrons in their outermost layer and are relatively reactive, close to alkaline earth metals. The empty *d* orbitals in these elements make them highly susceptible to becoming binding sites and providing effective doping sites for oxides. Kim et al. reported the method of a rapid flame doping method (40s) for introducing cobalt dopant into TiO_2_ (Co–TiO_2_) which formed cobalt dopant‐oxygen vacancy pairs and hence reduced Ti^3+^ trap states.^[^ ^119^ ^]^ CoCl_2_ was introduced into the precursor solution of SnO_2_, which simultaneously passivated the oxygen vacancies in SnO_2_ as well as the perovskite interface in contact with SnO_2_, thereby inhibiting charge recombination at the interface (**Figure** **7**a–c).^[^ ^120^ ^]^ Additionally, Co doping led to more favorable energy level arrangements for charge extraction. Finally, the device achieved an open‐circuit voltage of up to 1.2 V and a maximum efficiency of 23.82%.

Lanthanides have been a preferred choice for ETL dopants due to their unique electronic properties of 4*f* orbitals. The trivalent rare earth (RE) ions, such as Eu^3+^, Er^3+^, Sm^3+^, have been reported to modify the TiO_2_ to enhance the UV stability of the device. The introduction of RE ion dopants with well‐shielded 4*f*‐orbital configurations could functionalize TiO_2_ as down‐shifting phosphors that could convert a single high‐energy photon to one low‐energy photon.^[^ ^121^, ^122^ ^]^ Chen et al. reported that Eu‐TiO_2_ effectively converted destructive UV photons into useful visible luminescence for additional light, and constructed a more desirable energy band alignment at the interface (Figure 7d,e).^[^ ^123^, ^124^ ^]^ The device efficiency based on Eu‐TiO_2_ ETL was improved to 21.40% from 19.22% for the control device. The Er‐doped TiO_2_ was able to reduce the concentration of oxygen vacancies, which may also account for the better stability of the device.^[^ ^122^ ^]^ La‐doped BaSnO_3_ (LBSO) had higher carrier mobility, better conductivity, and stability. Seong Sik Shin et al. reported a low‐temperature (300 °C) solution reaction method for the synthesis of lanthanum‐doped barium stannate ETL (Figure 7f,g).^[^ ^125^ ^]^ Compared with a TiO_2_‐based device with an efficiency of 19.7%, the LBSO‐based device achieved a stable efficiency of 21.2%, and the light stability of the device was substantially improved.

### 4.2 Element Doping for HTL

Doping MO‐HTLs also contributed to better energy band matching, higher conductivity and carrier mobility, lower defect density, and faster interface charge extraction.^[^ ^126^, ^127^, ^128^ ^]^ The use of Cd to dope NiO_*x*_ was found to be effective due to its similar ionic radius with Ni. The doping of Cd reduced the formation energy of Ni vacancy, resulting in a higher hole concentration in NiO_*x*_ and a shift in the Fermi energy level. The doping of Cd also lowered the VBM of NiO_*x*_, accelerated the extraction and transport of holes, and suppressed nonradiative recombination at the interface.^[^ ^129^ ^]^ As a result, the device efficiency was improved from 18.23% to 20.47%. Al was also used to dope NiO_*x*_, which reduced the roughness of the NiO_*x*_ film and enhanced the electrical conductivity. At the same time, the dipole layer formed at the interface further increased the carrier extraction efficiency.^[^ ^130^ ^]^ In addition, the precise control of surface doping and self‐doping effects on NiO_*x*_ was also emerging as a method for further breakthroughs in device efficiency.^[^ ^131^ ^]^ Du et al. devised a redox treatment for the preparation of NiO_*x*_ by electron beam evaporation, which solved the surface wetting problem. At the same time, this treatment greatly enhanced the electrical conductivity of the NiO_*x*_ layer, improved the energy band matching, passivated the defect states, and reduced the voltage loss of the device.^[^ ^131^ ^]^ These improvements allowed them to obtain the PSCs with an efficiency of 23.4% and the large‐area modules (156 × 156 mm^2^) with an efficiency of 18.6%.

## 5 Summary

The excellent photoelectric performance, good stability, variety of preparation methods, and wide range of application scenarios have made MO‐CTLs the most promising choices for industrial application in PSCs. Meanwhile, the tunable surface states, energy level structure, and morphology control also significantly contribute to the performance of PSCs. In this review, we summarized the preparation methods of MO‐CTLs and various strategies for improving their performance. Through morphology control, interface modification, and doping treatment, further breakthroughs in device performance can be obtained by enhancing the directional carrier transport, reducing surface defect states, improving interfacial energy level matching, interfacial contact, and stability.

Although various optimization strategies have been explored to address some of the issues faced by the MO‐CTLs, there are still additional factors that need to be considered in future research. First, the microscopic morphology of the CTLs needs to be more finely tuned to constitute carrier transport channels and networks that extend beyond nanorods or nanosheets. At the same time, surface immersion and stable contact as well as low defect states should be ensured. MO‐CTLs, particularly MO‐HTLs, have received relatively little attention in terms of morphology control. The modification of the surface groups via chemical bonding strength or molecular design could enhance the passivation action, interfacial compatibility, and stability. Another important area for future research is the doping of materials. Further investigation on the doping of materials should consider the selection of dopant elements and the changes they induce in the material. The reasonable selection of dopant elements requires careful consideration of their electronegativity, ionic radius, valence state, electron cloud structure, coordination polyhedron structure with oxygen, and crystal shape of the oxide of the doped element. Furthermore, significant modulation of semiconductor properties could also be achieved through precise induction of self‐doping and fine structural design.

## Conflict of Interest

The authors declare no conflict of interest.
